# The effects of financial incentives on diabetes prevention program attendance and weight loss among low-income patients: the We Can Prevent Diabetes cluster-randomized controlled trial

**DOI:** 10.1186/s12889-020-09683-5

**Published:** 2020-10-21

**Authors:** Jay R. Desai, Gabriela Vazquez-Benitez, Gretchen Taylor, Sara Johnson, Julie Anderson, Joyce E. Garrett, Todd Gilmer, Houa Vue-Her, Sarah Rinn, Katelyn Engel, Jeff Schiff, Patrick J. O’Connor

**Affiliations:** 1 HealthPartners Institute, Bloomington, MN USA; 2 Minnesota Department of Health, 85 East 7th Place, P.O. Box 64882, St. Paul, MN 55164 USA; 3 Minnesota Department of Human Services, St. Paul, MN USA; 4 University of California, La Jolla, San Diego, CA USA; 5 National Kidney Foundation, New York, NY USA

**Keywords:** Diabetes type 2 prevention, Lifestyle intervention, Financial incentives, Weight loss, Low income individuals, Medicaid

## Abstract

**Background:**

Penetration and participation of real life implementation of lifestyle change programs to prevent type 2 diabetes has been challenging. This is particularly so among low income individuals in the United States. The purpose of this study is to examine the effectiveness of financial incentives on attendance and weight loss among Medicaid beneficiaries participating in the 12-month Diabetes Prevention Program (DPP).

**Methods:**

This is a cluster-randomized controlled trial with two financial incentive study arms and an attention control study arm. Medicaid beneficiaries with prediabetes from 13 primary care clinics were randomly assigned to individually earned incentives (IND; 33 groups; *n* = 309), a hybrid of individual- and group-earned incentives (GRP; 30 groups; *n* = 259), and an attention control (AC; 30 groups; *n* = 279). Up to $520 in incentives could be earned for attaining attendance and weight loss goals over 12 months. Outcomes are percent weight loss from baseline, achieving 5% weight loss from baseline, and attending 75% of core and 75% of maintenance DPP sessions. Linear mixed models were used to examine weight change and attendance rates over the 16 weeks and 12 months.

**Results:**

The percent weight change at 16 weeks for the IND, GRP, and AC participants were similar, at − 2.6, − 3.1%, and − 3.4%, respectively. However, participants achieving 5% weight loss in the IND, GRP, and AC groups was 21.5, 24.0% (GRP vs AC, *P* < 0.05), and 15.2%. Attendance at 75% of the DPP core sessions was significantly higher among IND (60.8%, *P* < 0.001) and GRP (64.0%, *P* < 0.001) participants than among AC (38.6%) participants. Despite substantial attrition over time, attendance at 75% of the DPP maintenance sessions was also significantly higher among IND (23.0%, *P* < 0.001) and GRP (26.1%, *P* < 0.001) participants than among AC (11.0%) participants.

**Conclusions:**

Financial incentives can improve the proportion of Medicaid beneficiaries attending the 12-month DPP and achieving at least 5% weight loss.

**Trial registration:**

ClinicalTrials.govNCT02422420; retrospectively registered April 21, 2015.

**Supplementary information:**

**Supplementary information** accompanies this paper at 10.1186/s12889-020-09683-5.

## Background

An estimated 353 million adults worldwide are at high risk of developing diabetes [1]. Numerous randomized controlled diabetes prevention lifestyle interventions have demonstrated strong efficacy in delaying or reducing the onset of type 2 diabetes among high risk adults yet penetration and participation of real-world diabetes prevention programs has been limited [2–6]. In the United States this has been a particular challenge among low-income, less educated, and culturally diverse individuals [7, 8]. A meta-analysis of community-based DPP interventions and evaluation of the Centers for Disease Control and Prevention’s (CDC’s) National Diabetes Prevention Program (NDPP) found that program attendance and weight loss goals were much lower among black, Hispanic, and other racial/ethnic individuals compared to non-Hispanic whites [9, 10]. This is concerning because these individuals have poverty rates two times higher than those of non-Hispanic whites and, among those in the highest poverty level, diabetes prevalence is 18% compared to 8% for those in the lowest poverty level [11, 12]. Furthermore, low income individuals are increasingly insured through the public programs of Medicaid and Medicare, which account for 66% of the estimated $237 billion in annual direct medical costs attributable to diabetes in the United States [13, 14].

A potential solution is the use of extrinsic incentives to promote participation and outcomes in diabetes prevention lifestyle programs. Several studies have demonstrated that prompt and frequent delivery of incentives can lead to improved healthy behaviors [15–22]. Many insurers and 90% of large employers in the United States are using such incentives to foster healthy behaviors and there is a growing body of evidence that financial incentives improve tobacco cessation, physical activity, healthy eating, and weight management [16, 18–21, 23–25]. The 35 million economically disadvantaged adult Medicaid recipients may be especially responsive to financial incentives [21, 26]. Medicaid is a federal health care insurance program. In the United States adults at or below 138% of the federal poverty level are eligible for Medicaid. Some States, like Minnesota, have expanded insurance coverage to include adults at or below 200% of the federal poverty level. Currently, gaps remain in understanding the optimal incentive design for specific behaviors and populations, especially low income or culturally diverse populations who are not necessarily reached by corporate wellness programs.

At present, among 164 funded NDPP sites, 150 use participant incentives with 32 using financial incentives [5]. The NDPP incentive targets, how they are delivered and their effectiveness is, however, unknown. Providing incentives for DPP attendance may be a good approach considering that for every DPP session attended participants lost 0.31% of their baseline weight and, a 1 kg reduction in weight results in a 16% decrease in diabetes incidence [9, 27]. Providing incentives for attendance over 16 weeks was shown to be effective by VanEpps and colleagues as part of the Centers for Medicare & Medicaid Services Medicaid Incentives to Prevent Chronic Disease (MIPCD) initiative but it did not result in differential weight loss [28, 29]. The MIPCD was an ambitious effort to design and evaluate incentive programs that encourage smoking cessation, weight management, blood pressure and cholesterol control, optimal diabetes care management, and type 2 diabetes prevention [28].

We present findings from the We Can Prevent Diabetes (WCPD) study, also part of the MIPCD initiative [30]. The study was conducted among Minnesota Medicaid beneficiaries with prediabetes who were recruited from their primary care clinics. Two financial incentives structures were designed to offer immediate incentives for DPP attendance and weight loss over a 12 month period [30]. Our incentive design, patient population, recruitment process, and study duration differ from prior studies, thus adding to the current literature. We hypothesized that participants receiving financial incentives would improve DPP attendance and weight loss compared with participants not receiving incentives.

## Methods

### Study design

We conducted the three-arm, pragmatic cluster-randomized controlled WCPD study from January 2013 to December 2015 [30]. We compared Medicaid beneficiaries receiving the 12-month DPP for free but with no incentives for participation or weight loss (attention control; AC) with beneficiaries earning financial incentives based on their individual participation and weight loss (IND) and with beneficiaries earning incentives based on a hybrid of individual and group participation and weight loss (GRP). The WPCD study adheres to the CONSORT guidelines (see additional file). The study was approved by institutional review boards at the Minnesota Department of Health (primary), the Minnesota Department of Human Services, the University of Minnesota (on behalf of the University of Minnesota Physicians health system), HealthPartners, HealthEast, and Hennipin County Medical Center.

### Study participants and setting

Minnesota adults enrolled in Medical Assistance or MinnesotaCare (i.e., Medicaid) were identified, recruited, consented, and enrolled by clinic staff at 13 primary care clinic systems in the Minneapolis-St. Paul metropolitan area [30]. These clinics serve a culturally diverse, low-income population, including Hmong, Somali, Karen, and Latino immigrants. Participants were recruited through clinic visits, electronic medical record review with telephone outreach, and community-based outreach.

Eligible participants had to: a) be currently enrolled in Medicaid; b) be 18 to 74 years old; c) have a body mass index ≥25 kg/m^2^ (≥22 kg/m^2^ for those of Asian descent); and d) have prediabetes or a history of gestational diabetes mellitus (GDM). Prediabetes status was classified as: a) at least one elevated blood glucose reading within 18 months of enrollment (fasting plasma glucose 5.6–6.9 mmol/L [100–125 mg/dl]; impaired glucose tolerance 7.8–11.0 mmol/L [140–199 mg/dl]; or HbA1c 39–46 mmol/mol [5.7–6.4%]); or b) at least one outpatient visit with a diagnosis code indicating abnormal glucose levels (ICD-9-CM 790.21, 790.22, or 790.29). Previous GDM was determined by: a) at least one outpatient or inpatient ICD-9-CM 648.8x; b) any mention of GDM in the participants’ medical notes; or c) participant self-report of GDM [30]. Additional exclusion criteria were reported elsewhere [30].

### Randomization and interventions

Participants were assigned to a DPP group based on their preferred day, time, and location [30]. Groups were randomly assigned to a study arm 24 h before their first DPP core session using a computer-generated randomization sequence prepared by the study statistician. DPP lifestyle coaches accessed the group assignment and informed participants of their assignment at the first DPP core session.

All participants received, free of charge, the 12-month group-delivered DPP, based on the CDC NDPP standardized DPP curriculum [31]. The DPP core program consists of 16 weekly one-hour sessions. The DPP maintenance program consists of eight one-hour monthly “booster” meetings tailored to the needs of the DPP group. While participants remained in their randomized groups during the first 16 weeks, pragmatic delivery of the maintenance sessions allowed combining some participants into the same maintenance sessions. These participants retained their original incentive structures and were analyzed as such.

The DPP was delivered by YMCA lifestyle coaches [30]. During the study’s last year, trained primary care clinic staff also led 21 DPP groups but with maintenance sessions modified to every 2 weeks for 4 months. For accessibility, primary care clinics identified locations best suited to their patients for delivery of the DPP sessions. These were either on-site or at a nearby community center. When possible, native-speaking DPP lifestyle coaches or interpreters were used for non–English-speaking participants.

All participants received $25 for attending one of the first three group sessions as well as materials to support lifestyle change (i.e., measuring cups, food scale, transportation, childcare, and free access to facilities for physical activity). The IND and GRP participants could earn up to $520 [30]. IND participants received financial incentives for each session attended, for attending at least 75% of DPP core sessions, attending at least 75% of DPP maintenance sessions, and achieving 5, 7, *and* 10% weight loss during the core period and 5, 7%, *or* 10% weight loss by the end of the maintenance period [30]. GRP participants earned a financial incentive each time they attended a DPP session or achieved 5% weight loss during the core or maintenance periods. If their entire DPP group achieved 75% core session attendance, 75% maintenance session attendance, or 7% or 10% weight loss during the core period or by the end of the maintenance period, all members of that group received the financial incentive [30]. The detailed incentive structures are described elsewhere [30]. Weight loss targets were based on the NDPP requirements (5%), original DPP findings (7%), and a stretch goal (10%). If a participant dropped out due to loss of Medicaid eligibility (or other reasons described elsewhere), GRP incentives were based on the remaining DPP group participants [30]. Attending a make-up session counted as a regular session. Financial incentives were received on a reloadable debit card issued to all participants and automatically loaded when incentive goals were attained [30].

### Data collection

The lifestyle coaches collected participant attendance, weight, and weekly food and physical activity logs at each DPP core and maintenance session. Weight was obtained at the beginning of each session on a portable digital scale. Data were entered into the MyNetico system within 48 h of the session and automatically triggered the disbursement of earned incentives [30]. Clinic staff retrieved participant vital signs, laboratory data, and smoking status from their electronic medical records systems for baseline and 12-month follow-up. Medicaid claims data were used to determine prior cardiovascular disease (ICD-9-CM 410–414, 420, 421, 423–427, 429–438, 440–444, 447, 451–453, 557, 785, v42, v45, V53) and mental health conditions (ICD-9-CM 295–298, 300, 301, 309, 311). Participants received a $25 reimbursement for the 12-month follow-up visit.

### Outcomes

Our primary outcomes were mean weight change over the 16 core sessions and the 12-month DPP period. We also evaluated the proportion of participants who achieved 5, 7, and 10% weight loss at any core session and at their last attended maintenance session. Attendance outcomes included the mean number of core, maintenance, and total sessions attended; the percent of participants who attended 12 core sessions; and the percent who attended six maintenance sessions. We also reported percent completion of the food and physical activity logs, and the percent of participants reporting 150 min of activity per week [30].

### Statistical analysis

All analyses were based on a participants original assigned study arm. We used the chi-square and F-test to compare baseline differences between the study arms. Linear mixed models were used to examine weight change over the 16 weeks and 12 months for participants with two or more weight measurements within each time frame. To evaluate the effect of the financial incentives on weight change, pairwise contrasts between GRP and AC and between IND and AC were computed. We also examined contrasts between GRP and IND. The models included repeated weight measurements as the outcome variable; time (weeks from first measurement), incentive arm, a time-arm indicator, and baseline weight as fixed effects and random intercept and slope for groups, with an autoregressive AR (1) structure for the error term [30]. Weights were log transformed and then back-transformed to their original scale for comparison of study arms. We also conducted sensitivity analyses, accounting for weight trajectory outliers or over influential patterns.

We modeled attendance rates for DPP groups using a generalized linear model with binomial distribution and logit link with the same parametrization of the fixed effects [30]. A compound symmetry covariance for the error term was used to account for the nested structure of the data. For mean number of sessions attended, we used a linear mixed model, and for weight loss and attendance goals, we used a generalized linear model. Linear mixed models adequately handle missing data under the assumption of missing at random. A similar approach was used to examine completion of physical activity and food logs and self-reported physical activity during the first 16 weeks. All analyses were evaluated at a 0.05 significance level and conducted using SAS version 9.3 (SAS Institute, Inc., USA).

### Power analysis

A priori power calculations were estimated based on weight change and considered the randomization and delivery at the group level. Based on prior DPP studies, we estimated that IND and GRP participants would lose 6.8 kg (15 lbs) and 9.1 kg (20 lbs), respectively, or about 0.45 kg (1 lb) per week throughout the DPP Core Sessions. With 80% power, α_2_ = 0.05 (two-tailed test), an intraclass correlation coefficient (ICC) of 0.01, and a weight standard deviation (SD) of 19.3 kg (42.5 lbs), we expected to detect an effect size of 0.2 (3.9 kg; 8.5 lbs) between the incentive arms and the AC arm, with 44 groups of 10 participants per group [30].

## Results

From January 1, 2013 through April 15, 2016, the 13 WCPD primary care clinic systems identified 2871 potential participants, reaching 72% and enrolling and consenting 55.7% of those contacted (*n* = 1154) [30]. Of these, 43 participants were never assigned to a randomized class and 264 participants never attended a DPP session, with no significant differences between the study arms [30]. Our analyses included the remaining 847 participants who enrolled and attended at least one DPP session (73.4%). Ninety-three groups were started (IND = 33, GRP = 30, AC = 30) with, on average, nine participants per group.

Baseline characteristics of participants are detailed in Table 1. Participants were racially and ethnically diverse. Only 17% were White while 64% were African American. However, a large proportion of African Americans were Somali immigrants and whose primary language was Somali. Almost 54% had a BMI ≥35 kg/m^2^. Less than 15% had more than a high school education and 40% had evidence of a prior mental health condition.

**Table 1:** Baseline characteristics of the We Can Prevent Diabetes study subjects attending at least one Diabetes Prevention Program session, by study arm (*n* = 847; *IND* Individual; *GRP* Group/Individual; *AC* Attention Control)

Characteristics	IND (***N*** = 33; ***n*** = 309)	GRP (***N*** = 30; ***n*** = 259)	AC (***N*** = 30; ***n*** = 279)
**Glucose Entry Criteria (%)**			
HbA1c 39–46 mmol/mol (5.7–6.4%)	62.5	56.8	60.9
Fasting plasma glucose 5.6–6.9 mmol/L (100–125 mg/dl)	26.2	26.9	29.0
Impaired glucose tolerance: 7.8–11.0 mmol/L (140–199 mg/dL)	0.3	0	0.4
Abnormal glucose diagnosis code (ICD-9790.2x)	8.7	8.5	7.5
Self-report history of gestational diabetes	1.3	6.6	1.4
None documented	0.3	1.9	0.7
Mean baseline HbA1c (mmol/mol; SD)	40 (2) (5.8% (0.3))	40 (3) (5.8% (0.4))	40 (2) (5.8% (0.3))
Diabetes diagnosis code (ICD-9250.xx)	9.7	10.8	7.2
Baseline weight	96.4 kg (212.5 lb)	97.3 kg (214.5 lb)	97.1 kg (214.0 lb)
Mean baseline body mass index (kg/m^2^, SD)	35.6 (8.1)	37.6 (8.6)	36.6 (7.8)
**Female (%)**	70.9	72.6	70.2
**Mean Age (years, SD)**	48.6 (12.2)	47.4 (11.9)	48.9 (11.6)
**Race/Ethnicity (%)**			
White	15.5	25.1	10.8
Black/African American	70.6	54.0	64.5
Asian	3.9	2.3	6.4
American Indian/Alaska Native	5.2	10.0	15.4
Native Hawaiian/Pacific Islander	0	0	0
Hispanic/Latino	4.2	6.2	1.4
Other or missing	0.6	2.3	1.4
**Primary Language (%)**			
English	61.2	78.4	79.2
Spanish	2.9	3.5	0.4
Somali	31.7	15.4	14.3
Hmong	3.6	0.8	4.7
Other	0.6	1.9	1.4
**Education Level (%)**			
< High school	40.8	33.2	35.9
High school	49.2	50.2	51.2
More than high school	10.0	16.6	12.9
**Marital Status (%)**			
Never married	47.6	47.9	53.4
Married	19.4	13.1	11.8
Other	33.0	39.0	34.8
Previous cardiovascular disease^a^ (% Yes)	8.7	12.4	9.0
Previous mental health condition^a^ (% Yes)	37.9	40.9	41.2
YMCA lifestyle coach	79.3	86.5	81.7
Clinic-based lifestyle coach	20.7	13.5	18.3

a. Based on Medicaid claims ICD-9-CM diagnostic and procedure codes.

Weight Change Outcomes: Based on planned analyses, at 16 weeks the mean percent weight change was similar for the three study arms (IND = − 2.6% (95%CI -3.1, − 2.0); GRP = − 3.1% (95%CI: − 3.7,-2.4); AC = − 3.4% (95%CI: − 9.2, 4.9)) (Table 2). At 12 months, mean percent weight changes for IND, GRP, and AC participants were − 3.7% (95%CI: − 5.7,-1.6), − 4.4% (95%CI: − 6.5,-2.2), and − 7.1% (95%CI: − 9.2,4.9) and non-significant. However, these results reflect the differential attrition rates across groups, and thus the number of participants contributing with data in the maintenance phase: 142 (IND), 122 (GRP), and 71 (AC) participants with two or more weight measurements from week 17 to week 52.

**Table 2:** Primary outcomes in the We Can Prevent Diabetes study among participants attending at least one DPP session, by study arm (*n* = 847; *IND*Individual; *GRP* Group/Individual; *AC* Attention Control)

Primary Outcomes	IND (***N*** = 33; ***n*** = 309)	GRP (***N*** = 30; ***n*** = 259)	AC (***N*** = 30; ***n*** = 279)	IND vs AC difference	GRP vs AC difference
**Weight Change**^**a,b**^					
% Weight change at 16 weeks (95% CI)	−2.6 (−3.1, −2.0)	−3.1 (−3.7.-2.4)	−3.4 (−4.0,-2.7)	0.8, *P* = .06	0.3, *P* = .46
% Weight change at 12 months (95% CI)	−3.7 (−5.7,-1.6)	−4.4 (−6.5,-2.2)	−7.1 (−9.2, 4.9)	3.4, *P* = .47	2.7, *P* = .13
**Attendance**					
Mean number of DPP core sessions attended (95% CI)	11.1 (10.3, 11.9)	11.4 (10.5, 12.3)	10.5 (10.0, 11.0)	.58, *P* = .0005	.94, *P* = .0001
Mean number of ALL DPP sessions attended (95% CI)	13.6 (12.4, 14.8)	14.2 (12.9, 15.5)	12.7 (12.0,13.4)	.89, *P* = .0002	1.50, *P* < .0001

a. Number of individuals with two or more measurements for weight change at 16 weeks and from 17 to 52 weeks: IND = 279 and 142; GRP = 231 and 122; AC = 254 and 71.

b. The intra-class correlation coefficient for weight among the DPP groups was 0.11.

During DPP core sessions, the 5% weight loss goal was met by 15.2% of AC, 21.5% of IND, and 24.0% of GRP (*P* < 0.05) participants (Table 3). Significantly more GRP participants also attained 7% weight loss than did AC participants. At 12 months, significantly more IND (20.3%, *P* < 0.05) and GRP (21.5%, *P* < 0.05) participants achieved the 5% weight loss than did AC participants (14%). A greater proportion of GRP participants also achieved the 7% (*P* < 0.05) and 10% weight loss goals (*P* < 0.05) than did AC participants (Table 3). There were no weight loss goal differences between the IND and GRP participants. Additionally, among all participants achieving at least 5% weight loss, 80% maintained the weight loss through the 12-month intervention period (data not reported).

**Table 3:** Compliance metrics in the We Can Prevent Diabetes study among participants attending at least one session, by study arm.^a,b^ (*n* = 847; *IND* Individual; *GRP* Group/Individual; *AC* Attention Control)

Compliance Measures	IND (***N*** = 33; ***n*** = 309)	GRP (***N*** = 30; ***n*** = 259)	AC (***N*** = 30; ***n*** = 279)
***Achieved weight loss goal at any core session***			
Met 5% weight loss goal, %	21.5	24.0^*^	15.2
Met 7% weight loss goal, %	8.2	13.4^**^	9.3
Met 10% weight loss goal,%	2.3	4.3	1.5
***Achieve weight loss goal at any maintenance session***			
Met 5% weight loss goal,%	20.3^*^	21.5^*^	14
Met 7% weight loss goal,%	11.0	15.7^*^	9.7
Met 10% weight loss goal,%	4.9	10.3^*^	5.5
**Attendance (includes make-up sessions)**			
Attended at least 12 DPP core sessions, %	60.8^***^	64.0^***^	38.6
Attended at least six DPP maintenance sessions,%	23.0^***^	26.1^***^	11.0
**Food and Physical Activity Logs (weeks 4–16)**			
Mean number of sessions a participant completed their weekly food log	3.3	5.2^*^	3.4
Mean number of sessions a participant completed their weekly food log *per number of sessions attended*	0.42	0.63^*^	0.53
Mean number of sessions a participant completed their weekly physical activity log	5.0	5.5	4.5
Mean number of sessions a participant completed their weekly physical activity log *per number of sessions attended*	0.62	0.67	0.72
Mean physical activity minutes per week among participants reporting logs	164	213	177
Achieved at least 150 min per week, %	30.0	47.4*	35.6

a. Comparisons are between each incentive arm and the Attention Control arm.

b. *P < 0.05, **P < 0.01, ***P < 0.001

**Fig. 1:**
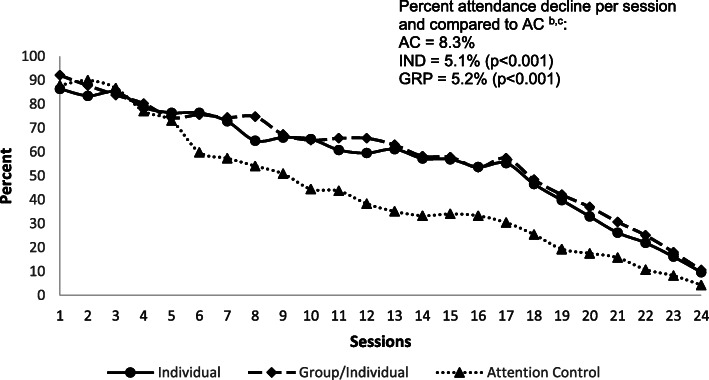
Attendance rates per Diabetes Prevention Program session^a^ (*n* = 847; IND = Individual; GRP = Group/Individual; AC = Attention Control). a Data on participants who attended at least one DPP session.b Attendance lines were fitted with a generalized linear model with each individual session as a categorical variable with a binomial link.c Difference in the percent decline was analyzed using a generalized linear model with session as a continuous variable

Attendance Outcomes: Attendance rates by session declined steadily from about 90% to just over 50% at week 16 in the IND and GRP participants. Among the AC participants, attendance was just over 30% at week 16. Rate of decline was similar for IND and GRP (*P* = 0.742) but were significantly lower compared to the AC participants (IND vs AC, *P* < 0.001; GRP vs AC, *P* < 0.001) (Fig. 1). Sixty-one percent of IND and 64% of the GRP participants attended at least 75% of the DPP core sessions, compared with 39% of AC participants (*P* < 0.001). Attendance of at least 75% of DPP maintenance sessions was 23 and 26% for the IND and GRP participants, respectively, compared with 11% for the AC participants (Table 3).

Food and Physical Activity Monitoring: Weekly completion of food and physical activity logs was monitored in DPP core sessions 4 through 16. In general, completion of food and physical activity logs was low with, on average, 3.3 to 5.2 sessions. This represented 42 to 63% of sessions attended, depending on the study arm. Among beneficiaries completing their physical activity log, the proportion self-reporting at least 150 min of physical activity per week was 30, 47, and 36% among the IND, GRP, and AC participants, respectively (Table 3).

Incentives earned: A total of $116,205 in incentives was disbursed to the 847 participants (IND = $60,120; GRP = $49,110; AC = $6975). The median, lower quartile, and upper quartile of incentives earned per participant for IND participants were $215, $85, and $285, respectively. These were $215, $95, and $270 for GRP participants and $25 for AC participants.

## Discussion

In this pragmatic randomized controlled trial with Medicaid beneficiaries in the United States, we found that financial incentives substantially increased DPP participation during core sessions and modestly increased participation during maintenance sessions. All three study arms lost 2.6 to 3.4% from their baseline weight (5.5 to 7.3 lbs.) during the first 16 weeks. By 12 months, weight loss was 7.1% in the AC study arm, compared with 3.7 and 4.4% in the IND and GRP study arms; however, due to participant attrition, these differences were not significant. Thus, financial incentives did not differentially affect mean weight loss during DPP core and maintenance sessions.

Despite no differences in mean weight loss, a greater proportion of IND and GRP participants attained 5% weight loss during the first 16 weeks and at 12 months than did AC participants. Significantly more GRP participants also achieved 7 and 10% weight loss at 12 months suggesting that group-based incentives may enhance weight loss by promoting group support or accountability. It was notable that sustained weight loss was observed among 80% of all participants who were able to achieve at least 5% weight loss. This may represent a high level of intrinsic motivation to improve healthy behaviors among these participants, potentially facilitated by extrinsic financial incentives since a greater proportion of participants in the IND and GRP groups achieved at least 5% weight loss.

Similar MIPCD DPP financial incentive interventions were implemented among Montana and New York Medicaid beneficiaries [28, 29, 32, 33]. A composite evaluation across all three studies found increased DPP attendance in participants receiving incentives but with no significant differences in weight loss between participants with and without incentives [33]. In contrast to the WCPD, New York and Montana did not find differences in the proportion of participants achieving 5% weight loss. In fact, in New York, 19 to 43% of participants across all study arms, including the 36% of the control group, achieved 5% weight loss [29, 32]. In all these studies, weight was only measured at a DPP session and thus reflects participants retained in the study over time. This is important to consider. Participant attrition at 16 weeks was almost two times higher in New York compared with WCPD, even with similar incentive structures [29]. This suggests other study design factors such as eligibility criteria, recruitment approaches, and DPP delivery may impact retention beyond the financial incentive structures and could potentially ‘wash-out’ incentive weight loss effects by retaining primarily participants with high initial intrinsic motivation in the treatment and control study arms.

Among Minnesota Medicaid beneficiaries, receipt of the incentives was immediate, and the GRP design may have fostered peer accountability and support. These design characteristics have proven effective in several weight loss and physical activity studies [19, 24, 34]. However, most previous studies lasted fewer than 6 months, and behavior changes were not sustained after the incentives were removed [15, 18, 34]. Longer-term (> 6 months) incentive effects are likely diminished due to loss of motivation, difficulty in adhering to the behaviors, and lack of or less frequent incentives [17, 23, 34–36]. We may have experienced this in the WCPD when, at 4 months, incentives changed from once a week to only once a month and attrition rates appeared to rise among the intervention arms.

The MIPCD studies used a standard-gain economic model for structuring incentives. Participants were guaranteed known financial rewards for participation, weight loss, and in Montana, completing food and physical activity logs. These are DPP leverage points within participant control. Future studies could explore other instrumental targets such as providing incentives for individually tailored step-wise improvements in healthy behaviors and outcomes. Another promising approach would be to explore incentive designs leveraging behavioral economic principles as these have demonstrated improved weight loss and physical activity compared to standard gain designs [15, 17, 18, 37]. Capitalizing on a person’s tendencies to expect future rewards based on past experience, desire for rapid feedback and rewards, aversion to loss, loss regret, and response to variable reinforcement may increase the effectiveness of financial incentives, particularly in low-income populations [22, 38, 39]. Currently, however, such studies in low-income or culturally diverse populations do not exist.

Participants in most DPP community-based translational studies have not been able to achieve a weight loss of 5% from baseline [9, 10, 40]. Furthermore, 25% of eligible NDPP participants did not even enroll in the program, and another 23% were lost to attrition [9, 10, 41]. We experienced similar issues, with 37% of those contacted declining to participate or never attending a DPP session after enrolling while among AC participants attending at least one DPP session, less than 40% attended 12 or more DPP core sessions, and only 11% attended 6 or more DPP maintenance sessions. Like the NDPP, the WPCD also found a 0.31% weight decrease for each attended session. But, even with the DPP provided at no cost, classes arranged for convenience (i.e., location, day, time, free childcare, and free transportation), cultural adaptations (i.e., culturally trained lifestyle coaches, interpreters, groups with culturally similar participants), and recruitment from trusted providers, sustained engagement among Medicaid beneficiaries without additional motivators like incentives was difficult [9].

Currently, eight States provide Medicaid DPP reimbursement. In the WCPD, accounting for programs costs and the incentives earned by Medicaid beneficiaries, the DPP was cost-effective and, therefore, should be more broadly considered by payers [42]. In addition, increasing organizational capacity to deliver the DPP in low-income communities is needed. This may require the CDC Diabetes Prevention Recognition Program to revisit the 5% weight loss recognition requirements; otherwise, DPP-delivery organizations serving low-income individuals will be in danger of losing their certification and reimbursement status, exacerbating diabetes health disparities [43, 44].

Limitations: Our study has some limitations. First, participants volunteered for this study and were mostly women, reducing generalizability to all Medicaid beneficiaries and suggesting novel strategies may be needed to engage men in the NDPP. However, from a pragmatic perspective, we believe Medicaid beneficiaries who are women would be more likely to participate in similar programs. Furthermore, our participants were racially, ethnically, and culturally diverse. They did, however, cluster within the same DPP groups which enhanced DPP delivery but reduced power for the analyses because the unit of randomization was the DPP group. In addition, we had initially powered on 44 DPP groups per arm with an average of 10 participants. At the end of the study, we had 30 groups per arm with an average of nine participants per group thus limiting our power to detect significant differences. Analytically, we offset the effect of these two factors by using repeated weight measurements within participants and covariate adjustment. Second, our primary outcomes were assessed at DPP sessions. If a participant did not attend a session, we did not have weight information, which was problematic, especially after 16 weeks. Third, though participant characteristics were associated with lost to follow-up these were similar across study arms, even though attrition was larger in the AC group. Overall, participants lost-to-follow-up at 16 weeks were younger, African American, had Somali as their primary language, and were less educated which suggests that future studies may need to tailor their designs to better engage and support participation of these individuals. Interestingly, even though 40% of participants had evidence of a mental health condition, this did not differentially impact attrition although it may have influenced adherence and weight loss. Finally, participants and lifestyle coaches were not blinded to the study assignments. Lifestyle coaches may have been more or less engaged given the intervention assignment, but we were unable to determine this potential effect. Nonetheless, all investigators, statisticians, and data analysts were blinded to study assignments until study completion.

## Conclusion

The DPP is an intensive, 12-month, structured lifestyle program designed to help participants increase physical activity, eat healthier, reduce caloric intake, lose weight, and ultimately, prevent or delay the onset of diabetes. Modifying habits established over many years is challenging—and potentially more so for Medicaid beneficiaries, who are often less educated, un- or under-employed, struggling to maintain family and housing stability, and living in obesogenic environments where food and physical activity options are limited [45]. Despite these difficulties, our study demonstrated that, with active outreach from trusted primary care providers, a culturally diverse group of Medicaid beneficiaries were interested in an intensive, structured, and free lifestyle behavior change program. However, reward-based financial incentives were needed to sustain participation and promote achievement of weight loss goals. Financial incentives may be an avenue to overcome individual, socioeconomic, and environmental barriers experienced by low-income populations in the United States and provide a model for bridging primary care health systems with community-based health programs [46]. We did find the WCPD to be cost-effective, thus, it could improve the current and future health status of a large number of Medicaid beneficiaries while mitigating future health care costs [42]. At the same time, future incentive studies need to carefully consider design factors such as individually tailored goals, targeting other outcomes such as physical functioning, and using incentive structures informed by behavioral economics to maximize participation and improvements in cardio metabolic outcomes among low-income populations that are quite heterogeneous in terms of race, ethnicity, immigration status, and culture.

### Key messages

Compared to no financial incentives, financial incentives can significantly improve attendance to diabetes prevention program sessions and may increase the proportion of participants achieving the goal of at least 5% weight loss from baseline weight.

## Supplementary information

**Additional file 1.**

## Data Availability

The datasets analyzed during the current study are available from the corresponding author upon request.

## Abbreviations

AC: Attention control group
CDC: Centers for disease control and prevention
DPP: Diabetes prevention program
GRP: Individual-group hybrid incentive group
IND: Individual incentive group
WCPD: We can prevent diabetes study
MIPCD: Medicaid incentives to prevent chronic disease initiative
